# The P2 Receptor Antagonist PPADS Supports Recovery from Experimental Stroke *In Vivo*

**DOI:** 10.1371/journal.pone.0019983

**Published:** 2011-05-17

**Authors:** Alexandra B. Lämmer, Alexander Beck, Benjamin Grummich, Annette Förschler, Thomas Krügel, Thomas Kahn, Dietmar Schneider, Peter Illes, Heike Franke, Ute Krügel

**Affiliations:** 1 Department of Neurology, Friedrich-Alexander-University of Erlangen-Nuremberg, Erlangen, Germany; 2 Department of Neurology, University of Leipzig, Leipzig, Germany; 3 Rudolf-Boehm-Institute of Pharmacology and Toxicology, University of Leipzig, Leipzig, Germany; 4 Department of Diagnostic and Interventional Radiology, University of Leipzig, Leipzig, Germany; McGill University, Canada

## Abstract

**Background:**

After ischemia of the CNS, extracellular adenosine 5′-triphosphate (ATP) can reach high concentrations due to cell damage and subsequent increase of membrane permeability. ATP may cause cellular degeneration and death, mediated by P2X and P2Y receptors.

**Methodology/Principal Findings:**

The effects of inhibition of P2 receptors by pyridoxalphosphate-6-azophenyl-2′,4′-disulphonic acid (PPADS) on electrophysiological, functional and morphological alterations in an ischemia model with permanent middle cerebral artery occlusion (MCAO) were investigated up to day 28. Spontaneously hypertensive rats received PPADS or vehicle intracerebroventricularly 15 minutes prior MCAO for up to 7 days. The functional recovery monitored by qEEG was improved by PPADS indicated by an accelerated recovery of ischemia-induced qEEG changes in the delta and alpha frequency bands along with a faster and sustained recovery of motor impairments. Whereas the functional improvements by PPADS were persistent at day 28, the infarct volume measured by magnetic resonance imaging and the amount of TUNEL-positive cells were significantly reduced by PPADS only until day 7. Further, by immunohistochemistry and confocal laser scanning microscopy, we identified both neurons and astrocytes as TUNEL-positive after MCAO.

**Conclusion:**

The persistent beneficial effect of PPADS on the functional parameters without differences in the late (day 28) infarct size and apoptosis suggests that the early inhibition of P2 receptors might be favourable for the maintenance or early reconstruction of neuronal connectivity in the periinfarct area after ischemic incidents.

## Introduction

Ischemic stroke is a leading cause for severe chronic morbidity or mortality in humans. Therapeutic strategies are limited and beside thrombolytic therapy [1] there are only few promising neuroprotective approaches at present (i.e. therapeutic hypothermia) [2]. Failure of energy-delivering processes after interruption of brain blood supply results in disturbances of essential ionic gradients, excessive neuronal depolarization releasing large amounts of excitotoxic compounds into the extracellular space, and consequently in cell death via necrosis or apoptotic cascades reflected in impaired sensory, motor and/or cognitive functions [3], [4]. Therefore, minimizing the devastating metabolic consequences of ischemia in brain and improving the recovery of function are current goals of therapeutic efforts.

Under physiological conditions neurons and astrocytes release purines into the extracellular space, involved in intercellular communication between neurons and astrocytes *via* activation of nucleotide receptors of the P2X- (ligand-gated ion channels) and the P2Y-subtype (G-protein coupled receptors) [5]–[7].

Under pathological conditions of acute mechanical injury or ischemia, purine nucleotides, stored at high millimolar range in the cytoplasm and synaptic vesicles, leak from damaged cells and may reach cytotoxic levels in the extracellular space in vitro [8], [9] and in vivo after experimental ischemia [10] and brain trauma [11]. These high extracellular nucleotide concentrations may activate several ATP/ADP- (and UTP/UDP-) sensitive receptor-types and their respective signal transduction cascades on various cell types [12]. All together, these P2 receptors may contribute in different magnitudes and time courses to the functional impairments observed after ischemia. Primarily, the stimulation of P2X receptors causes Ca^2+^ influx and can stimulate distinct Ca^2+^-dependent signalling cascades, which may have particular propensity to elicit cell death [13] e.g., by ischemia-induced excitotoxic glutamate release [14], or the activation of extracellular signal-regulated protein kinase (ERK) by P2X2 receptors [15]. P2X7 receptor-channels are thought to dilate in the presence of high ATP concentrations, forming large membrane pores of up to 900 Dalton size [16], [17], and inducing membrane blebbing and cytoskeletal reorganization [18], [19]. The activation of P2X7 receptors may lead to the release of proinflammatory cytokines like IL-1β [20] and the tumor necrosis factor-α [21]. Further, the stimulation of G protein-coupled P2Y receptors may trigger cell death by promoting the release of glutamate from nerve terminals and/or astrocytes [22] shown *in vivo* or by the activation of early apoptotic enzymes e.g., active caspase 3 via P2Y1 receptors [23].

Based on these findings it can be assumed that the partial blockade of P2 receptors diminishes the harmful consequences of ischemia induced ATP effects. By inhibition of P2 receptors, a protective effect on ATP-induced striatal injury was demonstrated; both, the extent of cell death and the lesion size were decreased [24]. After spinal cord injury, the wide spectrum P2 receptor antagonist pyridoxalphosphate-6-azophenyl-2′,4′-disulfonic acid (PPADS) and the P2X7 antagonist adenosine 5′-triphosphate-2′,3′-dialdehyde (oxATP) reduced the histological dimensions and functional sequelae in the peritraumatic zone [25]. The administration of PPADS not only accelerated the recovery of quantitative electroencephalogram (qEEG) in the acute and early post-phase of mechanical rat brain injury [26] but also reduced the concentration of extracellular glutamate [22]. Another non-selective P2 and glutamate receptor antagonist, suramin given prior to permanent focal cerebral ischemia, decreased the infarct volume six hours later [27]. In a previous study, we found that PPADS reduced the histologically estimated infarct area after the acute phase of ischemia after permanent middle cerebral artery occlusion (MCAO) [28]. Further, the recovery of motor, but not significantly that of cognitive disturbances ameliorated, under the treatment with PPADS for seven days, an effect, which was accompanied by a reduced amount of profoundly damaged cells [28].

The aim of the present study was to investigate, whether the early treatment of MCAO-induced ischemia with PPADS is beneficial for the late functional (behavioural) outcome in rats after 28 days. Because of the diversity of P2 receptors, which mediate a variety of harmful effects of high extracellular ATP concentrations, PPADS as a wide range P2 receptor inhibitor was used. Due to the early onset of stimulation of a number of P2X/Y receptors by ischemia-induced ATP/ADP release, a very fast intervention seems to be necessary. Therefore, though it does not reflect the clinical situation, the study was performed as a proof of principle, by beginning the antagonist treatment just before artery occlusion. Because primary inflammatory mechanisms can last for about one week, the treatment was maintained for seven days.

The periinfarct area is the target most likely rescued by pharmacological intervention or exercise [29]–[31]. Therefore, we investigated its neuroelectrophysiological activity measured as qEEG in parallel to the reconstitution of motor capabilities. Additionally, to follow up whether the difference in the infarct size in the acute phase persists after treatment, its volume was measured as a longitudinal and three-dimensional assessment by magnetic resonance imaging (MRI) non-invasively [32]. The imaging analysis was accompanied by the estimation of cell death in the periinfarct area in brain slices stained with terminal deoxynucleotidyl transferase-mediated dUTP nick end labelling (TUNEL), which indicates fragmented genomic DNA in situ. Finally, immunohistochemical studies with TUNEL method were performed to find out, whether both, neurones and astrocytes, underwent cell death after MCAO-induced ischemia.

## Materials and Methods

### Animals

Male spontaneously hypertensive rats (SHR; 250–300 g, Charles River, Bad Sulzfeld, Germany) were housed under a 12-hour light-dark cycle and allowed access to lab feed and water *ad libitum*. After the surgery, all animals were housed individually. All procedures were approved by the committee of Animal Care and Use of the relevant local governmental body in accordance with the law of experimental animal protection.

### Drugs

Drugs used for anaesthesia were ketamine hydrochloride (Ketanest®; Ratiopharm, Ulm, Germany) and xylazine hydrochloride (Rompun®; Bayer, Leverkusen, Germany). Artificial cerebrospinal fluid (ACSF; NaCl 126 mM, KCl 2.5 mM, NaH_2_PO_4_ 1.2 mM, MgCl_2_ 1.3 mM and CaCl_2_ 2.4 mM, pH 7.4) was used as control and vehicle for pyridoxalphosphate-6-azophenyl-2′,4′-disulphonic acid (PPADS; 1 µM, Biotrend, Köln, Germany). Both were administered for 7 days intracerebroventricularly by osmotic mini pumps (Alzet® Type 2001; total volume 200 µl, flow rate 1 µl/h, Charles River) with a total dose of 168 pmol PPADS/animal. The use of mini pumps allows a continuous drug administration and reduces the stress for animals induced by repeated manipulation for microinfusion. The *in situ* cell death detection Kit (terminal deoxynucleotidyl transferase-mediated dUTP nick end labelling, TUNEL) from Roche Diagnostics GmbH (Mannheim, Germany) was used and 3,3′-diaminobenzidine (DAB) was purchased from Sigma (Deisenhofen, Germany).

### Study design

**Figure 1:**
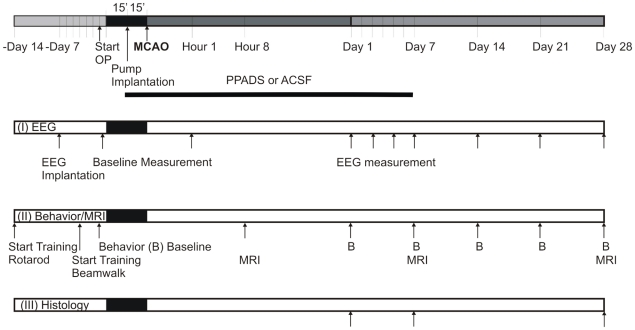
Time schedule of the treatments and investigations in the three types of experiments performed. OP, operation; MCAO, medial cerebral artery occlusion.

62 animals were assigned to three experimental groups schematically presented in Fig. 1. Two additional animals underwent MCAO for qualitative immunohistochemistry. All tests were performed by investigators blind to the treatment groups. At day 28, the animals were sacrificed and hemorrhagic transformation, subarachnoidal bleeding, or dislocation of the brain kit was excluded by histological examination. One animal of the ACSF group assigned to the EEG studies died at day 1 after MCAO. Another animal of the PPADS group assigned to the behavioural tests showed an intracerebral bleeding in the MRI and was replaced by another one.

- *Electroencephalography (EEG):* 16 animals were implanted with EEG electrodes and were subjected to MCAO 7 days afterwards. The animals were treated either with ACSF (n = 8) or PPADS (n = 8) for up to 7 days as described below. The individual EEGs were repeatedly recorded at day 3, 5, 7, 14 and 21 until day 28 after MCAO as indicated in Fig. 1.
- *Behavioural tests and MRI:* Behavioural training for rotarod and beam walk started 14 and 4 days prior to MCAO, respectively, with 16 rats. The animals were randomly assigned to two groups treated either with ACSF or PPADS (n = 8 each) and underwent MRI investigations after 8 hours and at days 7 and 28 after surgery. Additionally, the motor tests were performed at day 1, 7, 14, 21, and 28 after MCAO with these animals.
- *Histology:* 30 animals, assigned to either the ACSF or PPADS group, received the MCAO. The animals of both treatment groups were randomly assigned to survive until day 1, 7 or 28 (n = 5 each).

### Implantations and MCAO

**Figure 2:**
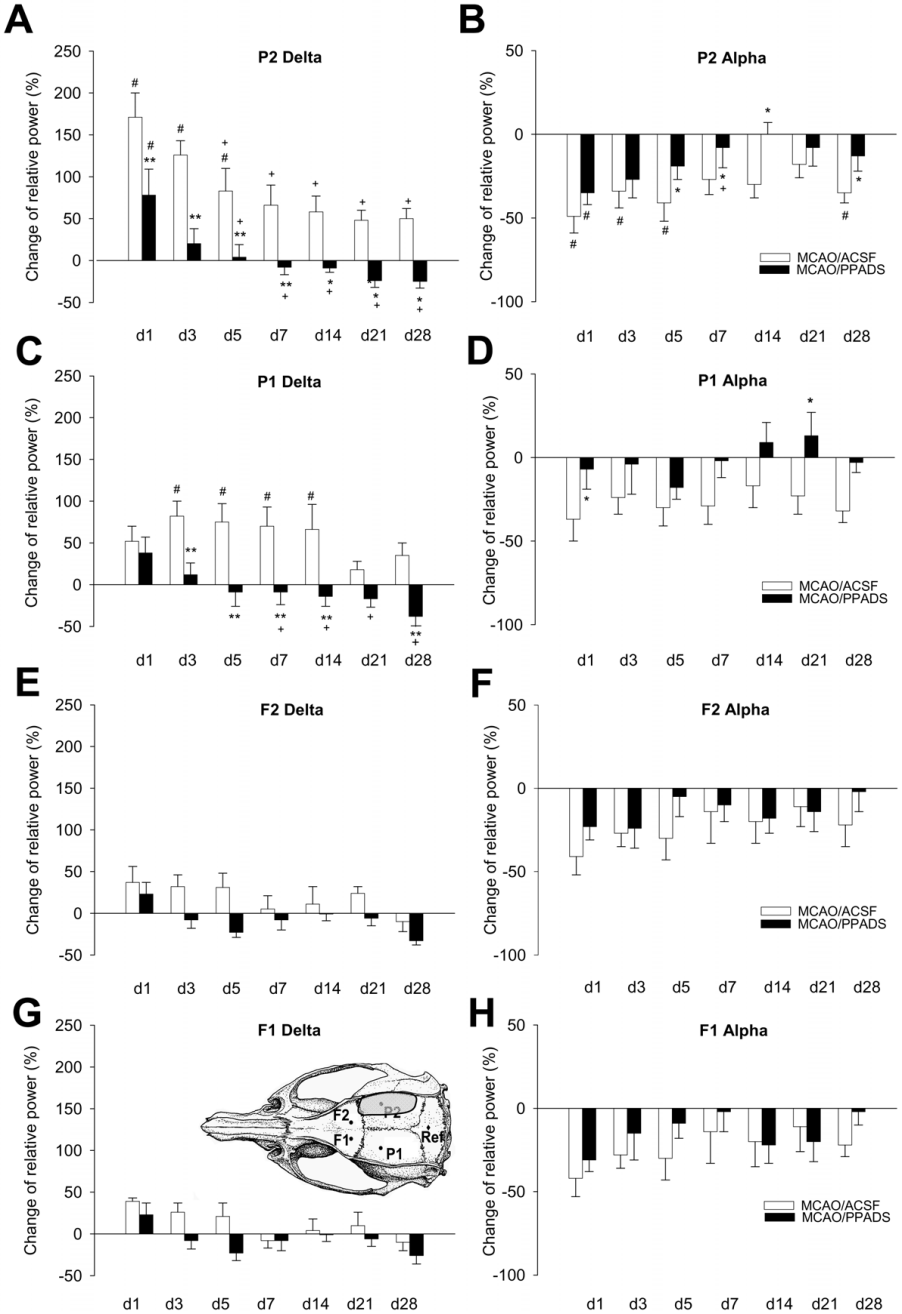
Effect of PPADS on the time course of EEG changes in the delta- and alpha-frequency bands caused by permanent MCAO of rats in four cortical regions up to day 28 after permanent MCAO. The delta-frequency band is shown in the left panel (**A**, **C**, **E**, **G**) and the alpha-frequency band in the right panel (**B**, **D**, **F**, **H**). The inset shows the placement of the four cortical electrodes and the reference electrode. The electrode P2 is located above the expected ischemic area indicated by grey shading, whereas the other electrodes are located above the contralateral parietal cortex (P1) and the respective frontal cortices (F2 and F1). Data are calculated as percental changes of the relative power from baseline measurements and expressed as means ± SEM. (ACSF: n = 7; PPADS: n = 8), * *P*<0.05, ** *P*<0.001 *vs.* MCAO/ACSF; + *P*<0.05 *vs.* day 1, # *P*<0.01 *vs.* basal.

Implantations and MCAO were performed under anaesthesia with ketamine hydrochloride (100 mg/kg, intramuscularly) and xylazine hydrochloride (5 mg/kg, intramuscularly). During surgery, the body temperature was maintained at 37°C with a heating pad and controlled with a rectal thermometer. The implantation of electrodes was performed at day 7 before MCAO. Five stainless steel screws were implanted as surface electrodes positioned on the surface of both hemispheres as indicated in Fig. 2G: frontal at AP +1.5 mm and L ±3.5 mm for the left (channel F1) and right hemisphere (channel F2) and parietal at AP −4.5 mm and L ±4 mm for the left (channel P1) and right hemispheres (channel P2, above the expected infarct area) [33]. The fifth screw (reference) was positioned at AP +11 mm and L 0 mm. The electrodes were soldered with a socket (TSE, Bad Homburg, Germany) and fixed with dental cement at the skull.

Osmotic pumps were filled with either ACSF or PPADS, conditioned for 14 hours at 37°C in sterile saline solution, and afterwards connected with a rat brain infusion kit (Alzet®, Charles River). The infusion kit was implanted into the right cerebral ventricle at coordinates relative to bregma: AP −1.0 mm, L +1.5 mm and V 4 mm below the dura and fixed with dental cement at the skull. The osmotic pump was connected to the brain kit and placed at the back of the rat subcutaneously. Immediately, after fixation of the osmotic mini pump the MCAO was performed as previously described [28]. Briefly, the right middle cerebral artery was prepared *via* a transtemporal approach. After retraction of the temporalis muscle, a 3 mm burr hole was drilled rostral to the fusion of zygoma and squamosal bone. After opening and retracting the dura mater, the middle cerebral artery was elevated by a steel hook, moved *via* a micromanipulator, and was electrocauterized. This last step was performed approximately 15 minutes after the implantation of the osmotic pump. The incision was suture closed. At day 8 after MCAO all pumps were removed under anaesthesia.

### Telemetric EEG recording

For EEG recording, the rats were transferred in their home cages to the experimental room and were allowed to adapt for 30 minutes. The EEG was recorded for 15 minutes at the same time of each examination day. To monitor the qEEG of the freely moving rats, a telemetric system (TSE, Bad Homburg, Germany) was used [34]. A transmitter was fixed at the electrode socket by plug connection. The EEG signals were telemetrically transferred *via* pulse position modulation and were transmitted to a receiver. The data acquisition was coupled online to a computer that transformed the data into real time means of fast Fourier analysis and displayed them as continuous spectra of power density. The obtained global spectra were divided into four frequency bands (Hz). 0.5–4.0 Hz (δ-band), 4.0–8.0 Hz (θ-band), 8.0–13.0 Hz (α-band) and 13.0–30.0 Hz (β-band). The sampling rate of the digitized EEG signals was 128/second. The EEG data were analyzed and presented as the percent change of relative power of the δ- and α-bands, indicating the relationship between EEG amplitude and frequency and detecting shifts of the EEG power among the selected frequency bands in comparison to prior the MCAO.

Simultaneously, the analogue signal and the behaviour of the animal were recorded for artefact detection (SigmaPLpro with videometry; SIGMA Medizin-Technik, Thum, Germany). The individual baseline EEG was recorded one hour prior to the MCAO surgery and served as reference. Further recordings were collected at days 1, 3, 5, 7, 14, 21 and 28 *post* MCAO once daily.

### Motor function

**Figure 3:**
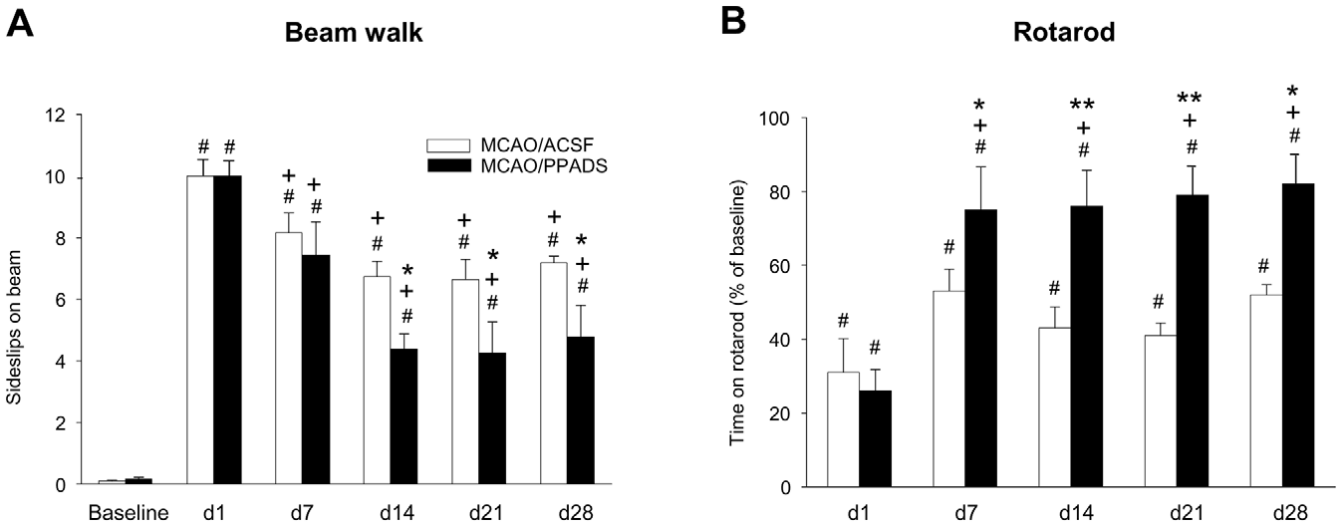
Effect of PPADS on the motor deficit in the beam balance test and the rotarod test up to day 28 after permanent MCAO. Values are expressed as means ± SEM. (n = 8 each group) of sideslips on the beam (**A**) and the percentage time of baseline the animals kept on the rotarod (**B**), * *P*<0.05, ** p<0.001 *vs.* MCAO/ACSF, + *P*<0.05 *vs.* day 1, # *P*<0.01 *vs.* basal.

The beam walk and rotarod tests were performed in the morning and the afternoon, respectively, to ensure an adequate recovery period between both tests. Training started once daily for the rotarod test at day 14 before and that for the beam balance test at day 4 before MCAO. At day 1 before MCAO, the time the rats remained running at the rotarod was set as individual baseline. After the MCAO both tests were performed at days 1, 7, 14, 21 and 28 (Fig. 3).

#### Beam walk test

A wooden beam 130 cm long and 15 mm wide was elevated 40 cm above the floor. The elevated home cage of each rat was positioned at one end of the beam. The rats were placed on the far end of the beam and trained to walk the beam toward their home cage. The test distance started at 30 cm from the near end and was 100 cm at all. To quantify motor deficits, the time to cross the beam and the number of hind foot slips from the beam were recorded from 5 repetitive trials intermitted by a recovery period of 15 minutes after each trial. In case of incompetence to walk on the beam and falling down, the number of sideslips was set to 10. The data are presented as the mean of 5 trials/per animal.

#### Rotarod

To avoid overlooking less severe deficits, motor function was proved at an accelerated rotarod (TSE). The animals were placed on the wheel rotating with 6 rounds per minute (rpm) and the time the animals remained on the accelerating wheel up to 40 rpm within 8 minutes was measured. The test was repeated 3 times with a recovery interval between the trials of 30 minutes. The data are presented as percentage of the mean remaining time on the wheel (3 trials/animal) compared to baseline.

### Magnetic resonance imaging (MRI) and volumetric image analysis

First MRI measurements were performed 8 hours after MCAO as diffusion weighted imaging (DWI), representing the cytotoxic oedema from the earliest phase of ischemia, but overestimating the infarct volume at later stages, and as T2-weighted imaging (T2W) showing the interstitial oedema. The second and third measurement was performed as T2W at days 7 and 28 after MCAO, representing tissue necrosis in this later phase. All measurements were carried out under anaesthesia with ketamine. MRI was performed with a 1.5T Philips Gyroscan Intera human whole body spectrometer using a small loop RF-coil (47 mm; Microscopy coil, Philips, Hamburg, Germany). The animals were monitored *via* the pneumatic respiration sensor of the MR scanner. Identical MRI parameters were used. T2W was acquired with a 163×208 matrix (MD); a 50×50 mm^2^ field of view (FOV), a slice thickness (SIT) = 1 mm, number of slices (NSl) measured = 20, with a turbo spin echo sequence (echo time [TE]/repetition time [TR] = 100/2051 ms and number of excitations [NE] = 8). In the first session, the animals underwent DWI with the following parameters: FOV = 50 mm, MD = 76×76, SlT = 1 mm and NSl = 12, measured with a T2 turbo-spin echo sequence (TE/TR) = 2800/40 ms and NE = 8 to diminish susceptibility artefacts. To determine the infarct volume, the T2W and DWI images were imported to the imaging program ImageJ 7.34s (Wayne Rasband, NIH, Bethesda, MD, USA). The affected regions were manually drawn on each slice to obtain the infarct area in mm^2^ and to calculate the volume by the formula: V_infarct_ = ∑ A_1_, A_2_, …, A_n_ [mm^2^]×thickness of slices [mm]. At days 7 and 28 after MCAO the post-necrotic cavity was partially connected to the lateral ventricle. Therefore, the size of the lesion including the ipsilateral ventricle was measured. The infarct volume was estimated by subtraction of the contralateral ventricle size as previously described [35].

### Histology

Animals were transcardially perfused at day 1, 7 or 28 after MCAO under thiopental sodium-anaesthesia with paraformaldehyde (2%) in sodium acetate buffer (pH 6.5) followed by paraformaldehyde (2%)/glutaraldehyde (0.1%) in sodium borate buffer (pH 8.5). Serial coronal sections (50 µm) containing the infarct area were collected as free-floating slices in 0.1 M Tris buffered saline (TBS, 0.05 M; pH 7.6). Terminal deoxynucleotidyl transferase-mediated dUTP nick end labelling (TUNEL) indicating fragmented genomic DNA *in situ* was performed according to the manufacturer's protocol. Briefly, brain sections were mounted onto gelatine-coated slides, permeabilized with 0.1% Triton X-100 in phosphate buffered saline (PBS, pH 7.4), and washed with PBS. Free 3′-OH termini of DNA strand breaks, generated by DNase preferentially during apoptotic cell death, and were labelled by incorporation of fluorescein-dUTP by terminal transferase reaction. The incorporated fluorescein was detected by anti-fluorescein antibody Fab-fragments conjugated with horseradish peroxidase *via* 3,3′-diaminobenzidine reaction. For negative control, permeabilized slices were incubated without terminal transaminase. The number of TUNEL-positive cells was counted within three arbitrarily chosen squares of identical size (0.25 mm^2^ each) positioned in the caudal and ventral periinfarct area of slices corresponding to bregma 1.5 mm (striatal level) and −3.2 mm (hippocampal level) using a light microscope (Axioskop; Zeiss, Oberkochen, Germany) with a 20× objective in connection with a laboratory cell counter (UZG1; Medical Academy, Magdeburg, Germany).

### Immunohistochemistry and confocal laser scanning microscopy

According to the manufacturer's protocol, TUNEL immunostaining was also performed as fluorescence labelling procedure in two animals, 7 days after MCAO [36]. Briefly, after washing with Tris-buffered saline (TBS, 0.05 M, pH 7.4), the slices with the fluorescein-labelled DNA strand breaks were exposed to TBS containing 0.3% Triton X-100 and 5% foetal calf serum (FCS) for 30 min for permeabilization and blocking. For the double labelling, the slices were then incubated with antibodies against glial fibrillary acidic protein (rabbit anti-GFAP, 1∶500; DakoCytomation, Glostrup, Denmark), or microtubule associated protein-2 (rabbit anti-MAP2; 1∶200; Chemicon, Temecula, CA, USA). Following intensive washing, the incubation with the secondary antibody Cy3-conjugated donkey anti-rabbit IgG (1∶1000; Jackson ImmunoResearch, West Grove, PA, USA) in TBS containing 0.3% Triton X-100 and 5% FCS for 2 hours at room temperature was performed. After intensive washing in TBS, the slices were processed through a 70, 90, 100% ethanol series, and finally n-butylacetate; then they were covered with entellan (Merck, Darmstadt, Germany). Finally, the reaction products were distinguished by their different fluorescence, analyzed by confocal laser scanning microscopy (LSM 510 Meta, Zeiss), using excitation wavelengths of 543 nm (helium/neon1, red Cy3-immunofluorescence) and 488 nm (argon, yellow-green Cy2-immunofluorescence, fluorescein).

### Statistical analysis

All data are presented as means ± SEM per group with the required sample size pre-estimated for the respective experimental study design using SigmaStat 3.5 (Systat Software GmbH, Erkrath, Germany). To determine statistically significant differences between the treatment groups and the daily differences between and within the groups in the behavioural tests, in the MRI and EEG, a two-way analysis of variance (ANOVA) with repeated measures was used. Differences in the number of TUNEL-positive cells were proved by ANOVA on Ranks with Dunn's Method. The Student-Newman-Keuls-Test was used *post hoc*. The level of statistical significance was set at *P*<0.05.

## Results

### Quantitative EEG spectral analysis

The MCAO induced in both the ipsi- (P2) and contralateral (P1) parietal recordings a marked slowing of EEG frequencies indicated by the increase of the power in the δ-band and a reduction in the α-frequency band (Fig. 2). Whereas these effects in the vehicle-treated group persisted until day 28, the EEG of PPADS-treated animals nearly recovered within the observation period. The frontal recordings were less affected by the ischemia.

The detailed cortical qEEG analysis of the ipsilateral P2 channel showed a significant acceleration of the reconstitution of the EEG spectrum after PPADS treatment compared to ACSF controls. The changes of power in the δ-frequency and α-frequency band (Fig. 2A, B) were significantly different in the ACSF and PPADS treated group (δ: *F*_1,110_ = 32.380; *P*<0.001 and α: *F*_1,110_ = 6.344; *P* = 0.022) and the EEG changes depended on the time after MCAO (δ: *F*_1,110_ = 13.563; *P*<0.001 and α: *F*_1,110_ = 6.344; *P*<0.001); however no significant interaction between treatment and day was found for the both frequency bands in P2.

Similarly, the contralateral P1 channel recovered faster after PPADS treatment in the δ- (*F*_1,110_ = 20.693; *P*<0.001) and in the α-frequency bands (*F*_1,110_ = 8.449; *P* = 0.011). The effects depended on the day after MCAO for the δ-band (*F*_1,110_ = 4.875; *P*<0.001) but not for the α-band (*F*_1,110_ = 1.626; *P* = 0.137) (Fig. 2C, D). There was a significant interaction between treatment and day in the δ-band (*F*_1,110_ = 3.614; *P* = 0.002) but not in the α-band (*F*_1,110_ = 0.709; *P* = 0.664). In both frontal recordings (channels F1 and F2; Fig. 2E–H) the EEG spectra were not detectably altered by treatment with PPADS.

Qualitative analysis of EEG revealed no signs of seizures at all, but in one animal seizure-like elements as singular spikes at days 14 and 21 were observed (not shown).

### Motor function

Ischemia induced impairments in motor functions (hemiplegia and hemiparesis) in both the ACSF- and PPADS-treated groups, which performed the task on the beam walk within 1.85±0.13 seconds with a minimum of foot slips (0.098±0.0285) on the day before the MCAO (pooled data). On the first day after MCAO, the animals were seriously impaired in their performance on the beam or rotarod, without differences between the treatment groups. At the following days, the treatment of MCAO-injured animals with PPADS improved the recovery of motor impairments in a statistically significant manner, when compared with the ACSF-treated controls, although there was no complete restitution to the baseline values. The recovery from MCAO-induced motor impairments on the beam was statistically significant for the days of treatment (*F*_1,79_ = 71,880; *P*<0,001). The number of foot slips on the beam was reduced by PPADS treatment at day 14 and later only (*F*_1,79_ = 6.186; *P* = 0.035) and there was a significant interaction between treatment and day (*F*_1,79_ = 2.936; *P* = 0.022) (Fig. 3A). In contrast to the number of sideslips, the time to cross the beam was not significantly altered by the treatments (not shown).

Further, PPADS prolonged the time the animals remained on the rotarod when compared with the ACSF-treated group (*F*_1,79_ = 24.197; *P*<0.001) (Fig. 3B). The improvement of the motor behaviour was statistically significant for all days after MCAO (*F*_1,79_ = 10.151; *P*<0,001) and for the interaction between treatment and day (*F*_1,79_ = 2.804; *P* = 0.019).

### MRI

**Figure 4:**
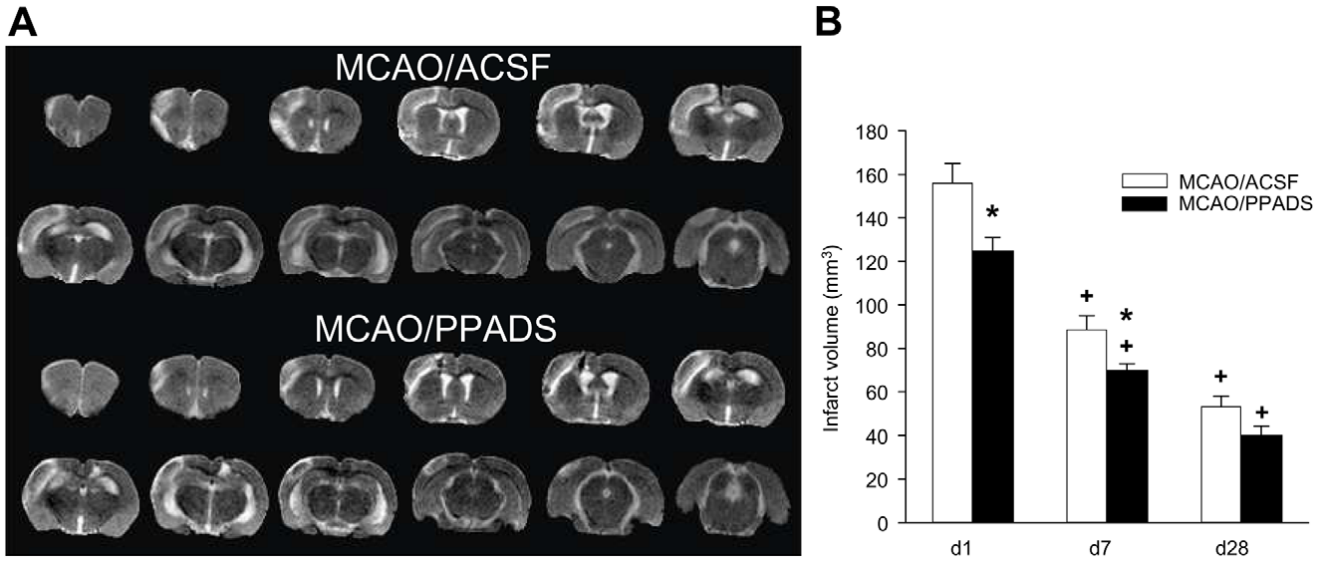
Effect of PPADS on the infarct volume calculated at days one, seven and 28 after MCAO from volumetric image analysis of T2W MRI. Examples for the extension of the infarct area at day 7 after MCAO with ACSF and PPADS by T2W MRI (**A**). Volumes are expressed as means ± SEM (n = 8); * *P*<0.05 *vs.* MCAO/ACSF (**B**). The infarct volumes are expressed as means ± SEM (n = 8 each), * *P*<0.05 *vs.* MCAO/ACSF treated animals, + *P*<0.05 *vs.* day 1.

After permanent MCAO, the ischemic damage was visible in the DWI (not shown) and T2W 8 hours after MCAO and in the T2W at days 7 and 28. The affected area mainly included the frontal and sensorimotor cortex and parts of the visual cortex according to previous histological studies [28], [36], [37] (Fig. 4A). The analysis of MRI data revealed a significantly faster reduction of infarct volumes by the treatment with PPADS (*F*_1,31_ = 12,192; *P* = 0,001) and a general reduction dependent on the day of measurement (*F*_1,31_ = 108,838; *P*<0,001) but no interaction between treatment and day. The differences of infarct volumes between the PPADS and ACSF group were significant at days 1 and 7 after MCAO, but not at day 28 (Fig. 4B).

### Histology/Immunohistochemistry

**Figure 5:**
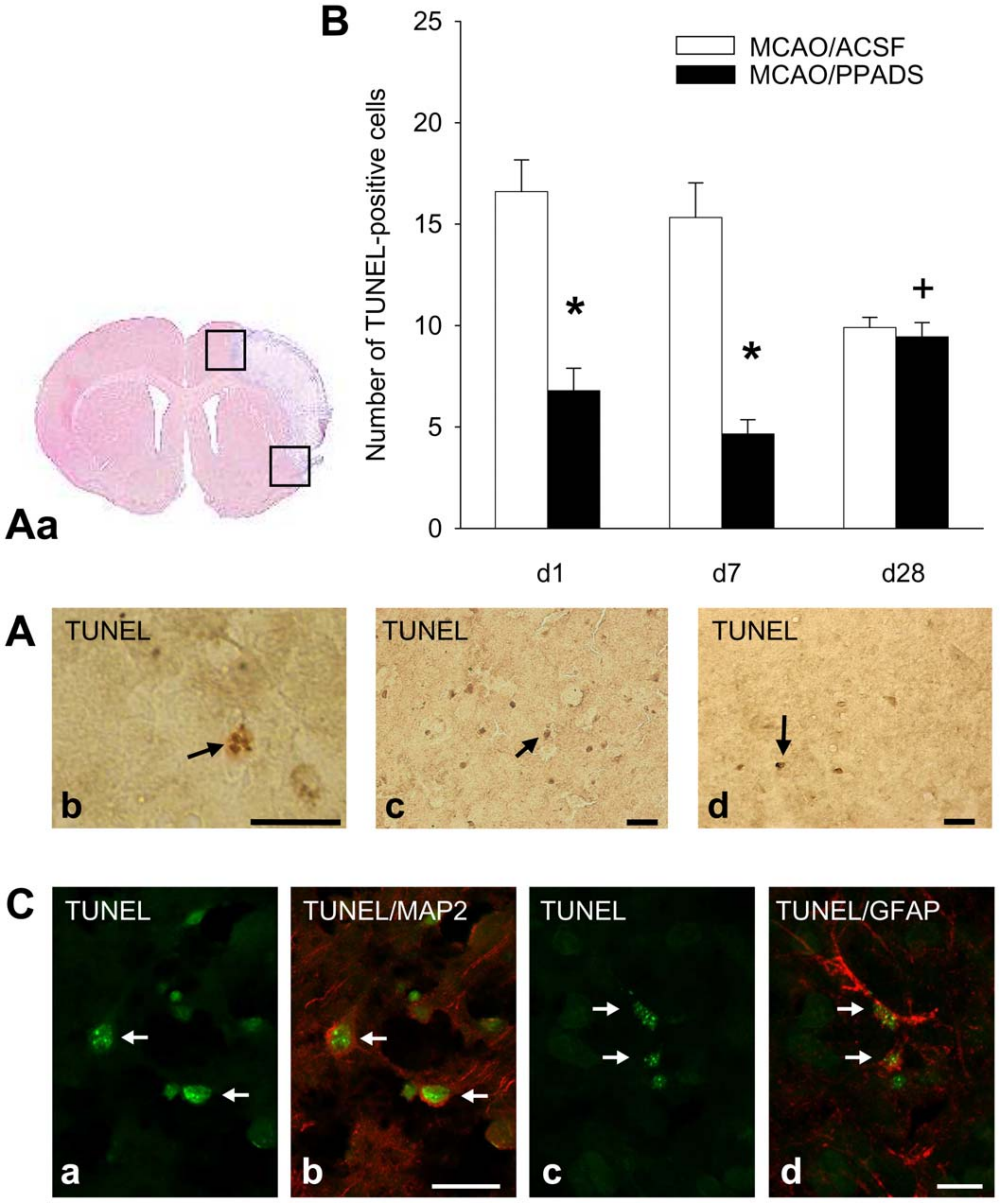
Effect of PPADS on the extent of cell death measured at TUNEL-positive cells. (**Aa**) The brain slice shows the infarct area at the striatal level of the brain and the caudal and ventral areas in the penumbra counted for TUNEL-positive cells. An example for a cell with deep brown-labelled apoptotic bodies (arrow) is given (**Ab**; scale bar: 20 µm). Images of caudal areas at day seven after MCAO of TUNEL-immunostained brain slices from animals treated either with ACSF (**Ac**) or with PPADS (**Ad**). TUNEL-positive cells are indicated by arrows. (**B**) The pooled number of TUNEL-positive cells counted on striatal and hippocampal brain slices from animals treated either with ACSF or with PPADS is shown for day 1, day 7 and day 28. * *P*<0.05 *vs.* MCAO/ACSF treated animals, + *P*<0.05 *vs.* day 7. (**Ca–d**) Confocal images of double immunofluorescence to characterize TUNEL-positive cell bodies (yellow-green immunofluorescence) in degenerating neurons (**b**, red Cy3-immunofluorescence, MAP2-positive), and in astrocytes (**d**, red Cy3-immunofluorescence, GFAP-positive) in the periinfarct area, seven days after MCAO. The arrows indicate co-expression in the same cell (Scale bars: (a,b) = 20 µm; (c,d) = 10 µm).

TUNEL-positive cells were histochemically detectable at days 1, 7 and 28 after MCAO (Fig. 5A,B). Statistically significant effects by treatment with PPADS were found (*H* = 32.639) (Fig. 5B). The appearance of TUNEL-positive cells in the PPADS group was reduced at days 1 and 7 (*P*<0.05) in comparison to the ACSF group at the striatal (Fig. 5A) and hippocampal section levels (not shown). Because there were no differences in the caudal and ventral areas of the penumbra, these data were pooled in Fig. 5B. However, after completion of drug administration at day 7, the amount of TUNEL-positive cells in the PPADS group was increased at day 28 (P<0.05) up to the amount found in the ACSF group. Further investigations with double labelling immunofluorescence showed that TUNEL-positive cell bodies in the periinfarct area belong to both, degenerating MAP2-positive neurones as well as to GFAP-positive astrocytes.

## Discussion

In this study, we have demonstrated that the inhibition of P2 receptors by PPADS improved the recovery of the cortical electrophysiological and motor functions from ischemia induced by permanent MCAO in rats. Furthermore, PPADS pre-treatment led to an initially decreased infarct volume and reduced cell death up to 7 days after MCAO compared to ACSF controls.

The permanent MCAO in rats mandatorily leads to a focal ischemia with neocortical infarcts followed by motor coordination deficits, which are most severe and reproducible regarding to the topography and volume of the infarct and consequently to functional impairments in the SHR strain in comparison to other normotensive strains [31], [38].

Permanent ischemia leads to energy depletion by limiting the delivery of oxygen and glucose to neurons, initiating excitotoxic mechanisms, which include among others the release of glutamate and ATP into the extracellular space, the subsequent activation of respective receptors, depolarization of the plasma membrane of neurons and increase of intracellular calcium, generating apoptosis and cell death [10], [39]. The penumbra around the infarct core, which receives limited blood supply from the collateral circulation, is affected by energy imbalance and is sensitive to extracellular stress and “danger” signals like ATP [40].

The P2 receptor antagonist PPADS is thought not to pass the intact blood brain barrier efficiently because of its polar structure. Therefore, PPADS was infused intracerebroventricularly for 7 days continuously at a concentration shown to be effective on EEG changes in a microdialysis approach of brain injury and in a previous study on the amount of profoundly damaged cells [26], [28].

Our data show, that P2 receptor blockade resulted in an accelerated recovery of ischemia-induced EEG changes of the delta and alpha activity in the ischemic and contralateral brain sides characterized by frequency slowing measured as a pronounced increase in the delta activity and a decrease in the alpha band in both parietal recordings. Such a slow down was reported to generally occur after stroke both in humans [41]–[43] and rodents [44], [45]. In the course of ischemia, the EEG power distribution recovered with a remaining focus near to the infarct core determined by the electrophysiological status of the surrounding penumbra [45]. In stroke patients, EEG plays a prominent role in monitoring of acute stroke and the recovery of the delta activity correlates well with the clinical outcome [43], [46].

In the present study, ischemia induced profound impairments in balance (vestibulomotor function) and motor coordination measured at the beam balance test [47] and on the accelerated rotarod, which is more sensitive to mild lesions [48]–[50]. The blockade of P2 receptors led to an improved motor deficit recovery in accordance with the faster rearrangement of the electrophysiological function in the PPADS treated group. These improvements of motor and qEEG function suggest that PPADS treatment protects cells against the consequences of ischemia-induced release of ATP and therefore leads to functional changes possibly also reflected by alterations of the infarct size.

In fact, the MRI investigations point to an initially smaller interstitial oedema by PPADS at day 1 and day 7. At day 28, when oedemas usually completely cleared, infarct core areas of nearly similar size remained, in which profoundly injured cells have died. The missing effect on the final size of the infarct area may have various reasons. Most likely, cells of the core area are not susceptible to PPADS treatment, because of the severity of the ischemia, which is still aggravated by a limited collateral circulation known to exist in this rat strain used. Thus, in the core area, cells are irreversibly damaged and cannot be rescued by any drug treatment. Moreover, though the image analysis is unable to supply any information about the functionality of the surviving periinfarct area, we suggest that an accelerated restitution of the initial infarct size by PPADS may reflect rather a protection than an improved functional reorganisation of the periinfarct area [30] measured as improved motor behaviour and EEG.

Previously, histological data on the reduction of the lesion size induced by spinal cord injury or MCAO in the acute phase up to 24 hours and at day 1 and 7 after, respectively, were presented [25], [28]. The present results are corroborated by data showing a reduced amount of reversibly and profoundly injured cells in favour of intact cells in the penumbra of PPADS-treated compared to ACSF-treated animals in the acute phase after MCAO [28].

Further confirming our findings, an improved hind limb motor recovery up to day 28 after spinal cord injury under treatment with PPADS and adenosine 5′-triphosphate-2′,3′-dialdehyde (oxATP; P2X7 receptor antagonist) accompanied by a diminished apoptotic cell death in the peritraumatic zone 24 hours after spinal cord injury was described [25]. Taken together, behavioural and electrophysiological results as well as morphological alterations *in vivo* are obviously related to a diminished cellular injury by treatment with PPADS.

In accordance, we found that PPADS obviously directly inhibited apoptotic processes during administration until day 7 as indicated by TUNEL visualized DNA fragmentation known to mark the end stage of irreversible cell damage [51]. Three weeks after finishing the PPADS treatment, the apoptosis rate has returned to the normal value with a time course similar to that determined in the ASCF-treated group. We speculate that the decrease in apoptosis, which typically peaks 24 hours or longer after stroke onset [52], [53], might be a sign for PPADS-mediated cell protection in acute ischemia. As we could show by double immunofluorescence staining, apoptosis is a common feature of cell death of neurons and astrocytes, with a close association with potentially salvageable tissue. Therefore apoptosis has attracted particular attention as a target for neuroprotective therapies [54].

PPADS acts as a slowly reversible or even irreversible antagonist, which is an advantage for the present approach, because the bioavailability of other, more selective P2 receptor ligands under in vivo conditions is unknown. PPADS acts at a variety of ligand-gated (P2X1, P2X2, P2X3, P2X5, and P2X7) and G-protein coupled (P2Y1 and P2Y4) receptor subtypes [5], [55]–[57], and might exert its supportive effects by more then only one mechanism.

One way of PPADS action may proceed via P2X7 receptors, which function as cation channels in the presence of low agonist concentrations. A prolonged stimulation with higher agonist concentrations can create non-selective membrane pores [16], or can stimulate multiple downstream signalling components, e.g. mitogen-activated protein kinases (MAPKs; ERK, p38, SAPK/JNK), Rho kinase and caspases leading to apoptosis/necrosis, and membrane blebbing, all involved in cell death [58].

Furthermore, PX7 receptor activation at immunocompetent cells, e.g. macrophages and microglia, evokes a strong inflammatory response [59]–[61], which is also a core event in ischemia and therefore in focus as a therapeutic target [62]. An up-regulation of P2X7 receptor protein on microglia cells was shown at day 1 and on neurons and astrocytes at day 4 after permanent MCAO in rats [36]. This was accompanied by the appearance of the apoptotic executioner active caspase 3 mediating cell death and by co-localization of the P2X7 receptors on TUNEL-positive apoptotic cells in the periinfarct area. Similarly, in a brain stab wound injury model [63] we demonstrated that various P2 receptor agonists, such as ADPβS (P2Y1,12,13), 2-MeSATP (P2X; P2Y1), and BzATP (P2X7) contribute to microglial activation. Furthermore, we showed that these effects were inhibited by the antagonists PPADS and Brilliant blue G (P2X7), suggesting that both, P2X and P2Y receptors might be involved in TNF-α, IL-1β and IL-6 release [20], [21], [64], [65], which are cytokines of microglial origin.

An up-regulation of P2X2 and P2X4 receptors in the hippocampus of gerbils in topographic and temporal accordance with ischemic cell death was found [66]. Whereas the intense expression of P2X4 receptors on microglia seems to be a compensatory mechanism, neuronal P2X2 receptors appear to be mechanistically involved in the cell death process. Given that an up-regulation of P2X receptors rather might reflect a higher demand of ATP signalling, it is unlikely that the recovery facilitating effects of PPADS are directly mediated *via* inhibition of these receptors.

Not only purine but also pyrimidine nucleotides are released during ischemia and prolonged activation of P2Y4 receptors by UTP induced cell death in human neuroblastoma cells, the early inhibition of these receptors could prevent the commitment to cell death [67].

P2Y1 receptor inhibition may also contribute to the beneficial effects of PPADS. This receptor mediates trophic effects [23] but also facilitates the endogenous release of glutamate inhibited by PPADS and the selective antagonist MRS 2179 *in vivo* which both have no direct effects on glutamate receptors [22]. After a mechanical microlesion of the brain, the elevation of extracellular ATP went along with a lasting elevation of glutamate [11]. Together, the acute injury-evoked stimulation of P2Y1 receptors may contribute to the glutamate-mediated excitotoxicity and functional impairments. Finally, in the above mentioned stab wound study, P2Y1 receptor activation enhanced the early apoptosis marker active caspase 3 in a PPADS reversible manner. However, pretreatment with PPADS prevented not only the P2Y1 receptor mediated raise of the apoptotic pERK1/2 but also of the anti-apoptotic pAkt [23] suggesting that P2Y1 receptors may contribute by multiple mechanisms and in concert with other signals time-dependently to death or survival of ischemia-affected cells. This is supported by the observation that the expression of P2Y1 receptors was enhanced seven days after mechanical injury [68] as a sign for an increased need/sensibility for nucleotide signalling subsequently to the initial phase high extracellular ATP and also counteracted by PPADS treatment.

All these data rise the following questions for future investigations: (1) Which time-window of treatment may be suitable to mimic the clinical scenario; (2) Which are the optimal antagonist doses being both efficient and tolerable; and (3) Will the use of more selective P2 receptor antagonists e.g., for P2X7 receptors, have any advantages in comparison with the non-selective P2 receptor antagonist PPADS for the long term treatment of ischemia.

In conclusion, though the morphological parameters, the infarct size, and the amount of apoptotic cells were diminished only up to day 7 by PPADS and did not differ significantly any more at day 28, the inhibition of P2 receptors in the early phase after ischemia improved the functional outcome within the observation interval of 28 days. This occurred probably by protection of the periinfarct tissue against P2 receptor stimulation by high extracellular nucleotide concentrations, rather than by promoting tissue reconstitution.
